# Sodium pyruvate exerts protective effects against cigarette smoke extract-induced ferroptosis in alveolar and bronchial epithelial cells through the GPX4/Nrf2 axis

**DOI:** 10.1186/s12950-023-00347-w

**Published:** 2023-08-21

**Authors:** Ziwen Zhao, Zhao Xu, Jingwen Chang, Liwei He, Zijin Zhang, Xiaoyu Song, Xianbang Hou, Fangtian Fan, Zhijun Jiang

**Affiliations:** 1 School of Pharmacy, Nanjing University of Chinese Medicine, 138 Xianlin Avenue, Nanjing 21, Taizhou, Jiangsu 0023 China; 2 Jiangsu Changtai Pharmaceutical Co., Ltd, Taizhou, Jiangsu 225300 China; 3 Anhui Engineering Technology Research Center of Biochemical Pharmaceuticals, School of Pharmacy, Bengbu Medical College, 2600 Donghai Avenue, Bengbu, Anhui 233003 China; 4 School of Pharmacy, Nanjing University of Chinese Medicine, Nanjing, 210023 China

**Keywords:** COPD, Sodium pyruvate, Ferroptosis, GPX4, Nrf2

## Abstract

**Background:**

Ferroptosis in alveolar and bronchial epithelial cells is one of the main mechanisms underlying the development of chronic obstructive pulmonary disease (COPD). Sodium pyruvate (NaPyr) is a natural antioxidant in the body, exhibiting anti-inflammatory and antioxidant activities. NaPyr has been used in a Phase II clinical trial as a novel therapy for COPD; however, the mechanism underlying NaPyr-mediated therapeutic benefits in COPD is not well understood.

**Objective:**

We aimed to assess the protective effects of NaPyr and elucidate its potential mechanism in cigarette smoke extract (CSE)-induced ferroptosis.To minic the inflammatory response and ferroptosis triggered by cigarette smoke in COPD in an invitro cell based system, we expose a human bronchial epithelial cells to CSE.

**Methods:**

To minic the inflammatory response and ferroptosis triggered by cigarette smoke in COPD in an invitro cell based system, the A549 (human lung carcinoma epithelial cells) and BEAS-2B (bronchial epithelial cells) cell lines were cultured, followed by treatment with CSE. To measure cellular viability and iron levels, we determined the levels of malondialdehyde (MDA), glutathione (GSH), reactive oxygen species (ROS), mitochondrial superoxide (MitoSOX), membrane potential (MMP), and inflammatory factors (tumor necrosis factor [TNF] and interleukin [IL]-8), and examined CSE-induced pulmonary inflammation and ferroptosis. To clarify the molecular mechanisms of NaPyr in COPD therapy, we performed western blotting and real-time PCR (qPCR) to determine the expression of glutathione peroxidase 4 (GPX4), nuclear factor E2-related factor 2 (Nrf2), and cyclooxygenase 2 (COX2).

**Results:**

We found that NaPyr effectively mitigated CSE-induced apoptosis and improved apoptosis induced by erastin, a ferroptosis inducer. NaPyr significantly decreased iron and MDA levels and increased GSH levels in CSE-induced cells. Furthermore, NaPyr suppressed ferroptosis characteristics, such as decreased levels of ROS, MitoSOX, and MMP. NaPyr significantly increases the expression levels of GPX4 and Nrf2, indicating that activation of the GPX4/Nrf2 axis could inhibit ferroptosis in alveolar and bronchial epithelial cells. More importantly, NaPyr inhibited the secretion of downstream inflammatory factors, including TNF and IL-8, by decreasing COX2 expression levels to suppress CSE-induced inflammation.

**Conclusion:**

Accordingly, NaPyr could mitigate CSE-induced ferroptosis in alveolar and bronchial epithelial cells by activating the GPX4/Nrf2 axis and decreasing COX2 expression levels. In addition, NaPyr reduced the secretion of inflammatory factors (TNF and IL-8), affording a novel therapeutic candidate for COPD.

**Graphical Abstract:**

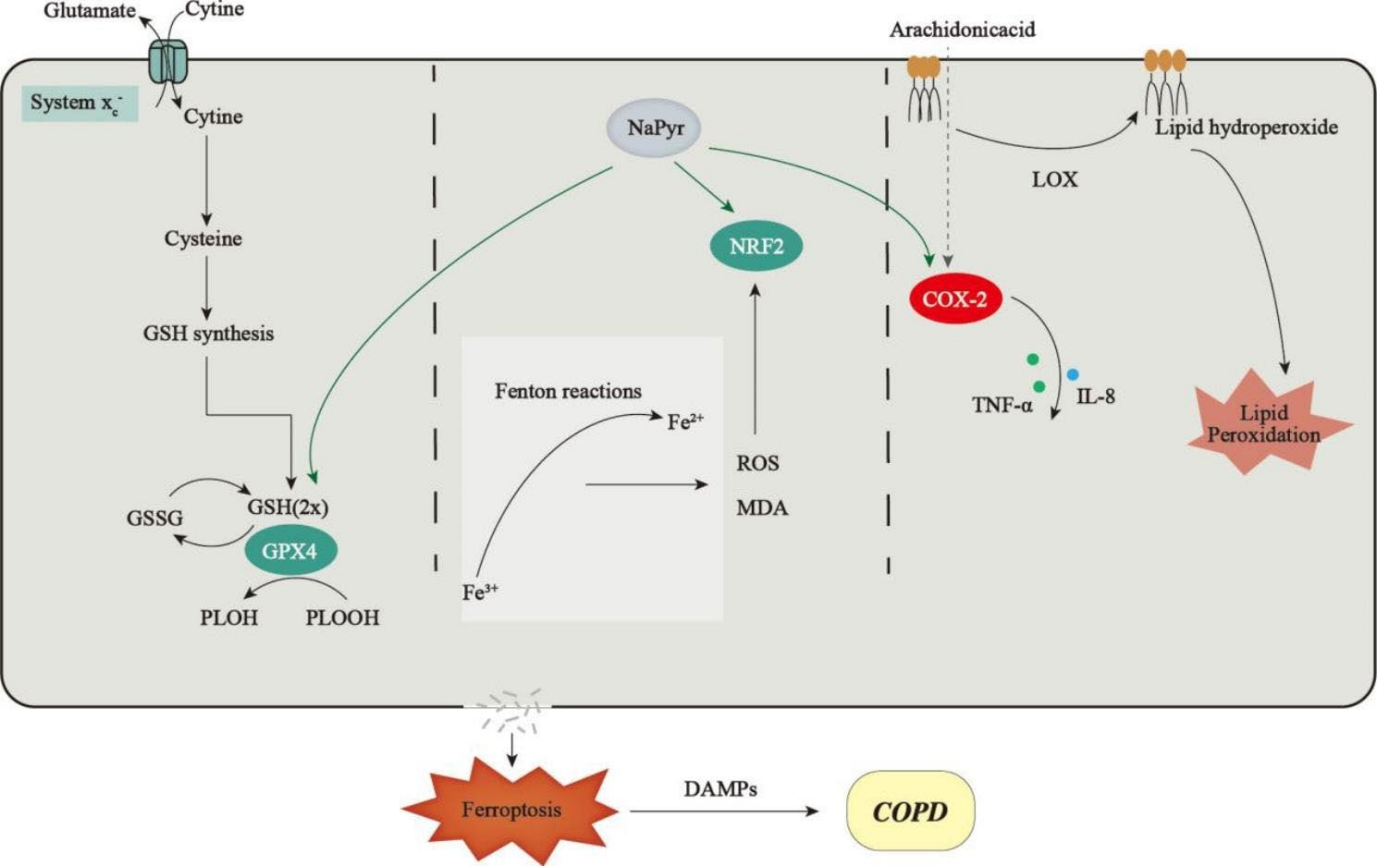

## Introduction

Chronic obstructive pulmonary disease (COPD), comprising chronic bronchitis and/or emphysema, is characterized by obstructed airflow from the lungs. Although COPD is a common chronic disease, it can progress to pulmonary heart disease and respiratory failure. According to data from World Health Organization (WHO), COPD is associated with substantial morbidity and mortality and is the third-leading cause of mortality worldwide [1–4]. Globally, it is estimated that approximately 384 million individuals suffer from COPD [5]. The onset of COPD has been associated with environmental factors and family history, and recent research has implicated inflammatory damage, oxidative stress, protease/anti-protease imbalance, and autoimmune responses. Among them, oxidative stress is one of the main causes of COPD, where an imbalance between oxidative and antioxidative processes increases reactive oxygen species (ROS)-induced damage in lung tissues directly or indirectly by inactivating the proteolytic pathway. Smoking is the most crucial risk factor implicated in the onset of COPD [6], and an increase in the smoking rate has been directly associated with the prevalence of COPD. Exposure to cigarette smoke is considered the main source of ROS in lung tissues. Oxidants in cigarette smoke, such as toxic particles and H_2_O_2_, have been associated with the incidence of COPD [7].

Ferroptosis, a recently discovered type of programmed cell death, is a non-apoptotic and necrotic form of cell death induced by disrupted synthesis and metabolism of biomolecules such as proteins, nucleic acids, and lipids, which was directly or indirectly caused by oxidative stress owing to ROS accumulation [8]. Ferroptosis has been closely linked to the incidence and development of multiple pulmonary diseases, including tuberculosis [9], lung cancer [10], and acute lung injury [11]. A recent study demonstrated that exposure to cigarette smoke extract (CSE) can induce unstable iron accumulation and increase lipid peroxidation in lung epithelial cells, along with non-apoptotic cell death [12]. Ferroptosis inhibitors such as desferrioxamine or ferrostatin-1 (Fer-1) could be used to treat lung epithelial cells and decrease CSE-induced lipid peroxidation. In addition, bronchial epithelial cells derived from healthy subjects and patients with COPD have been examined. Human bronchial epithelial cells (HBECs) derived from patients with COPD were found to exhibit changes in key regulators of ferroptosis, including glutathione peroxidase 4 (GPX4), nuclear factor E2-related factor 2 (Nrf2), and cyclooxygenase 2 (COX2), thereby suggesting that ferroptosis participates in the progression of COPD [12]. COX2 is a specific ferroptosis marker, and patients with COPD exhibit significantly elevated COX2 expression. GPX4 protein is a phospholipid-peroxylated glutathione peroxidase, shown to decrease the toxicity of lipid peroxidation products, maintain cell membrane-lipid bilayer stability, reduce GPX4 activity or expression, and induce intracellular lipid peroxide accumulation due to glutathione (GSH) depletion, and ultimately result in ferroptosis [13]. Furthermore, elevated ROS levels can induce Nrf2 activation during ferroptosis, inhibiting the production of ROS and improving antioxidant capabilities of cells. Exogenous antioxidants activate Nrf2 and eliminate ROS. Accordingly, antioxidants and antioxidant supplements could improve ferroptosis in COPD and could afford a novel treatment strategy for COPD.

Sodium pyruvate (NaPyr) is known to naturally exists in the human body, and exogenous NaPyr can exert anti-inflammatory and antioxidant activities. NaPyr can significantly decrease inflammatory cytokines and oxygen free radicals, including interleukin (IL)-6, IL-8, and hydrogen peroxide (H_2_O_2_) [14]. A NaPyr inhaler has been shown to afford therapeutic benefits in patients with COPD [15]. In addition, NaPyr could inhibit CSE-induced inflammatory effects in airway epithelial cells. Reportedly, 40–80% of patients with COPD exhibit nasal symptoms. NaPyr has been shown to reduce levels of ROS and inflammatory cytokines, and decrease the severity of rhinitis and nasal congestion in patients with allergic rhinitis, which could substantially improve clinical symptoms of COPD [16]. NaPyr is known to be a highly effective ROS scavenger [17, 18]. At a concentration exceeding 1 mM, NaPyr was found to suppress the cytotoxic effects of H_2_O_2,_ with a dose-dependent reduction in H_2_O_2_-induced ROS formation [19]. Liu et al. [20]demonstrated that NaPyr could increase the activity of complex III in the mitochondrial respiratory chain, activate the mitochondrial thioredoxin system, and upregulate manganese superoxide dismutase (Mn-SOD) post-severe burns to regulate the level of mitochondrial ROS in pancreatic islets. NaPyr can combat oxidative stress by increasing the concentration of reduced substances (e.g., glutathione) [21]. Moreover, another study examined the safety and COPD-mediated therapeutic effects of NaPyr, and revealed that NaPyr exhibited good tolerance after 4 weeks of administration and considerably improved symptoms in patients with COPD [22]. Therefore, NaPyr could afford clinical benefits against COPD. However, NaPyr-mediated effects and underlying mechanisms in COPD mitochondria of alveolar epithelial cells remain unexplored. NaPyr is currently undergoing assessment in a phase II clinical trial for development as a novel therapeutic drug. NaPyr induces multiple modes of action to improve COPD; however, the potential role of NaPyr in CSE-induced ferroptosis in alveolar and bronchial epithelial cells remains elusive. Therefore, in the present study, we aimed to explore the mechanism underlying NaPyr-induced ferroptosis suppression using A549 and BEAS-2B cells and elucidate the role of NaPyr in reducing the inflammatory in COPD.

## Materials and methods

### Cell strains

Human lung carcinoma epithelial A549 cells and BEAS-2B were purchased from the American Type Culture Collection (ATCC) cell bank (China).

### CSE preparation

CSE was prepared as previously described [12]. Briefly, the cigarette filter was removed, and one end of the cigarette (Jiangsu Zhongyan Industry Co., China) was connected to a Pasteur pipette containing 10 mL serum-free DMEM (Beijing Solarbio, China) and the other end was connected to a 20 mL syringe. Next, the piston was pulled back once every minute to draw 20 mL, which was held in place for 2 s. Thereafter, the glass bottle was agitated to dissolve the smoke until one cigarette was utilized completely. Each cigarette corresponded to 10 mL of culture media and was defined as 100% CSE. Subsequently, 0.22 μm filters were used to remove bacteria, followed by aliquoting and storage at − 80 °C. Prior to experimentation, samples were rapidly thawed and used within 30 min. Based on experimental grouping, culture media was used to dilute 100% CSE to the required concentrations.

### Cell model

Briefly, A549 and BEAS-2B cells were thawed and cultured in DMEM containing 10% FBS (Gibco, USA) at 37 °C in a 5% CO_2_ incubator. Media was replaced once daily. Then, log-phase cells were collected and assigned to groups based on pre-experimental results and previous reports. The grouping was as follows: control group: A549 and BEAS-2B cells were cultured using the conventional method; experimental group: cultured A549 and BEAS-2B cells were treated with CSE for 24 h; low-dose group: cultured A549 and BEAS-2B cells were pretreated with 0.5 mM NaPyr(Jiangsu Changtai Pharmaceutical Co., China) for 2 h, followed by CSE for 24 h; medium-dose group: cultured A549 and BEAS-2B cells were pretreated with 1 mM NaPyr for 2 h, followed by CSE for 24 h; high-dose group: cultured A549 and BEAS-2B cells were pretreated with 2 mM NaPyr for 2 h, followed by CSE for 24 h; inhibitor group, cultured A549 and BEAS-2B cells were treated with CSE and Fer-1 (Aladdin Biochemical Technology Co., China).

### CCK8 assay for cellular activity

Briefly, log-phase A549 and BEAS-2B cells were cultured in a 96-well plate (3 × 10^3^ each well) containing 100 µL DMEM and treated based on the established grouping. Subsequently, the supernatant was collected, and 100 µL DMEM containing 10% CCK8 (Dojindo Laboratories Co., Japan) solution was added. A microplate reader (Bio-Rad Co., USA) was used to measure the absorbance (Ab) value at 450 nm wavelength. Cell activity (%) = (Ab value of treated well – Ab value of blank well)/(Ab value of control well – Ab value of blank well) × 100.

### Measurement of intracellular free iron concentrations

Briefly, A549 and BEAS-2B cells were placed in culture plates and assigned to predetermined groups. Following treatment, cells were harvested, and the number of cells was calculated. A microplate reader was employed to measure the free iron concentrations according to the total iron assay kit (Elabscience, China).

### Measurement of MDA and GSH content to assess lipid peroxidation

Briefly, 100 µL of each cell supernatant was mixed with 400 µL of MDA working reagent (Beijing Solarbio, China). The mixture was incubated at 100 °C for 30 min, cooled on ice, and centrifuged at 10,000 *× g* for 10 min at 4 °C temperature. Subsequently, 200 µL of supernatant was placed in a 96-well plate, and the absorbance was measured at 532 nm. The MDA concentration was calculated based on the measured absorbance. Similarly, GSH content in each group was detected according to the instructions of the GSH assay kit (Beijing Solarbio, China).

### Measurement of MitoSOX to assess mitochondrial oxidation levels

Briefly, cells were seeded into 6-well plates and treated according to established experimental grouping. Cells were rapidly rinsed with Hanks’ balanced salt solution (Wabcan, China). Next, 5 µM mitochondrial probe MitoSOX (Thermo Fisher Scientific, USA) was added to plates, followed by incubation in the incubator for 10 min. After fixing with 40 g/L triformol, nuclei were stained with DAPI (Beijing Solarbio, China) for 10 min and rinsed three times with methanol. After sealing, blue and red fluorescence intensities were observed using the fluorescence microscope (Ghuin Co., Japan). Finally, MitoSOX fluorescence intensity was analyzed using ImageJ software (National Institutes of Health, Bethesda, MD), reflecting mitochondrial oxidation levels.

### Measurement of ROS to assess mitochondrial oxidation levels

Briefly, cells were seeded in a 6-well culture plate and treated according to established experimental grouping. After removal of the culture medium, 1 mL of 10 µmol/L DCFH-DA (KeyGEN BioTECH Co., China) was added to each well, and plates were incubated for 20 min. The cells were then washed with serum-free culture medium to remove the DCFH-DA not taken up by cells. After sealing, green intensity (excitation wavelength: 485 nm, emission wavelength: 530 nm) was observed using a fluorescence microscope and analyzed using ImageJ software, reflecting cellular oxidation levels.

### JC-1 assay for mitochondrial membrane potential

Briefly, cells were seeded in a 6-well culture plate and treated according to established experimental grouping. The cells were washed with phosphate-buffered saline, followed by the addition of 1 mL culture medium to each well. Subsequently, 1 mL JC-1 working stock (Beijing Solarbio, China) was added to each well and mixed thoroughly. The plates were incubated in an incubator at 37 °C for 20 min. After incubation, the supernatant was removed, and the cells were rinsed with JC-1 dye buffer two times. Then, 2 mL of cell culture medium was added. The cells were observed under a fluorescence microscope using red (excitation wavelength: 488 nm, emission wavelength: 590 nm) and green (excitation wavelength: 485 nm; emission wavelength: 530 nm) filters.

### ELISA

Briefly, cells were seeded in a 6-well culture plate at a concentration of.

600,000 per well and treated according to established experimental grouping. The supernatant of each group was collected after 24 h, followed by centrifugation at 2500 *× g* for 20 min. TNF and IL-8 expression levels were measured according to the ELISA assay kit instructions (Yi Fei Xue Biotechnology, China).

### Quantitative PCR (qPCR)

Briefly, total RNA was extracted using the TRIzol assay kit (KeyGEN BioTECH Co., China). cDNA was synthesized using the cDNA synthesis assay kit (Tiangen Biotech, China). qPCR was performed using SYBR-Green Supermix (Invitrogen Life Technologies). Corresponding expression levels were calculated using the 2^–∆∆Ct^ method and GAPDH as the internal control. The primers used are summarized as follows.

**Table:** 

Sequences	
Nrf2	F: 5′-AACACAAGAGCCCCTGTGTGGC-3′ R: 5′-TGCCCCTGAGATGGTGACAA-3′
GPX4	F: 5′-ACAAGAACGGCTGCGTGGTGAA-3′ R: 5′-GCCACACACTTGTGGAGCTAGA-3′
COX2	F: 5′-CTGGCGCTCAGCCATACAG-3′ R: 5′-CACCTCGGTTTTGACATGGGT-3′
GAPDH	F: 5′-GGTTGTCTCCTGCGACTTCA-3′, R: 5′-TGGTCCAGGGTTTCTTACTCC-3′

### Western blotting to measure protein expression

For each group of A549 and BEAS-2B cells, total protein was extracted using RIPA lysis buffer, and the total protein concentration was measured using the bicinchoninic acid (BCA) method. Then, 20 µg of protein was subjected to sodium dodecyl sulfate-polyacrylamide gel electrophoresis (SDS-PAGE). The protein was transferred to a polyvinylidene difluoride (PVDF) membrane by wet transfer and blocked in 50 g/L non-fat milk at room temperature for 2 h. Subsequently, the primary antibody was added (GPx4, Nrf2, COX2, and GAPDH [1:1000], respectively)(Cell Signaling Technology, China), and the membrane was incubated overnight at 4 °C. After washing, the membrane was incubated in a secondary HRP-conjugated antibody for 2 h. After washing thoroughly, the blots were developed with substrate solution (Pierce Biotechnology, USA), and the chemiluminescent imaging system was used to examine fluorescence (Bio-Rad Co., USA). Fluorescence intensity was analyzed using Image Lab software.

### Statistical analysis

Data analyses were performed in SPSS version 18.0 (IBM Corp., Chicago, IL, USA). All experiments were performed in duplicate and repeated at least 3 times. The results are presented as mean ± SD (standard deviation). The experimental data were assessed for a Gaussian distribution. Two-tailed unpaired Student’s t tests were applied for comparison of two normally distributed groups; comparisons between more than two normally distributed groups were made by one-way ANOVA followed by pairwise multiple comparison (Student-Newman-Keuls method, q-test). Differences were considered statistically significant at P < 0.05.

## Results

1. NaPyr reduces CSE-induced cell death in A549 and BEAS-2B cells.

**Fig. 1:**
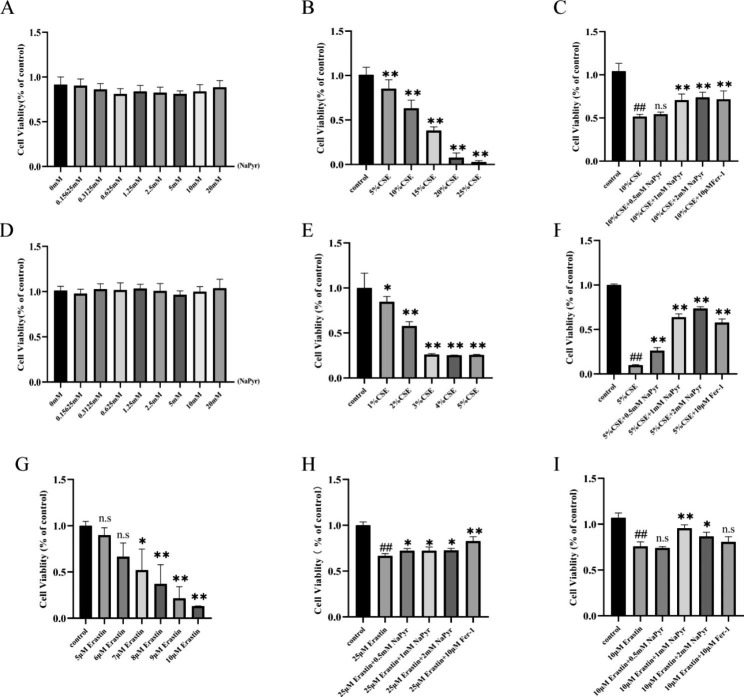
NaPyr reduces CSE-induced cell death in A549 and BEAS-2B cells. (**A**)Effect of NaPyr on cell proliferation of A549. (n = 6) (**B**) Changes of viablity in A549 after CSE stimulation. (n = 6; ^**^p < 0.01 vs. Control) (**C**) Changes of viablity in A549 after CSE stimulation and the intervention of NaPyr. (n = 6; ^##^P < 0.01 vs. Control; ^**^p < 0.01 vs. CSE) (**D**)Effect of NaPyr on cell proliferation of BEAS-2B. (n = 6) (**E**) Changes of viablity in BEAS-2B after CSE stimulation. (n = 6; ^*^P < 0.05; ^**^p < 0.01 vs. Control) (**F**) Changes of viablity in BEAS-2B after CSE stimulation and the intervention of NaPyr. (n = 6; ^##^P < 0.01 vs. Control;^**^p < 0.01 vs. CSE) (**G**) Cytotoxicity of Erastin to BEAS-2B. (n = 6; ^*^P < 0.05;^**^p < 0.01 vs. Control) (**H**) Changes of viablity in A549 after Erastin stimulation and the intervention of NaPyr. (n = 6; ^##^P < 0.01 vs. Control; ^*^P < 0.05,^**^p < 0.01 vs. CSE) (**I**) Changes of viablity in BEAS-2B after Erastin stimulation and the intervention of NaPyr. (n = 6; ##P < 0.01 vs. Control; ^*^P < 0.05,^**^p < 0.01 vs. CSE)

Using A549 and BEAS-2B cell lines, CSE-mediated cell models were developed to determine the role of NaPyr in COPD via anti-ferroptosis activity ex vivo. Based on the cell viability results, treatment with 0–20 mM NaPyr for 24 h did not impact cell proliferation (Fig. 1A and D), whereas CSE significantly decreased cell activity (Fig. 1B and E). Therefore, in subsequent cellular experiments, concentrations of 5% and 10% CSE were selected to stimulate A549 and BEAS-2B cells for 24 h. Furthermore, we demonstrated that the CSE-induced reduction in A549 and BEAS-2B cell viability could be alleviated by pretreatment with NaPyr and mitigated by the ferroptosis inhibitor Fer-1 (Fig. 1C and F), thereby indicating that CSE could induce ferroptosis. In addition, treatment with ferroptosis inducer erastin significantly decreased cell viability (Fig. 1G), and NaPyr administration could significantly ameliorate erastin-induced ferroptosis (Fig. 1H-I). Collectively, these results suggested that NaPyr could reduce ferroptosis and afford protection against CSE-induced cellular damage.

2. NaPyr inhibits CSE-induced ferroptosis in A549 cells.

**Fig. 2:**
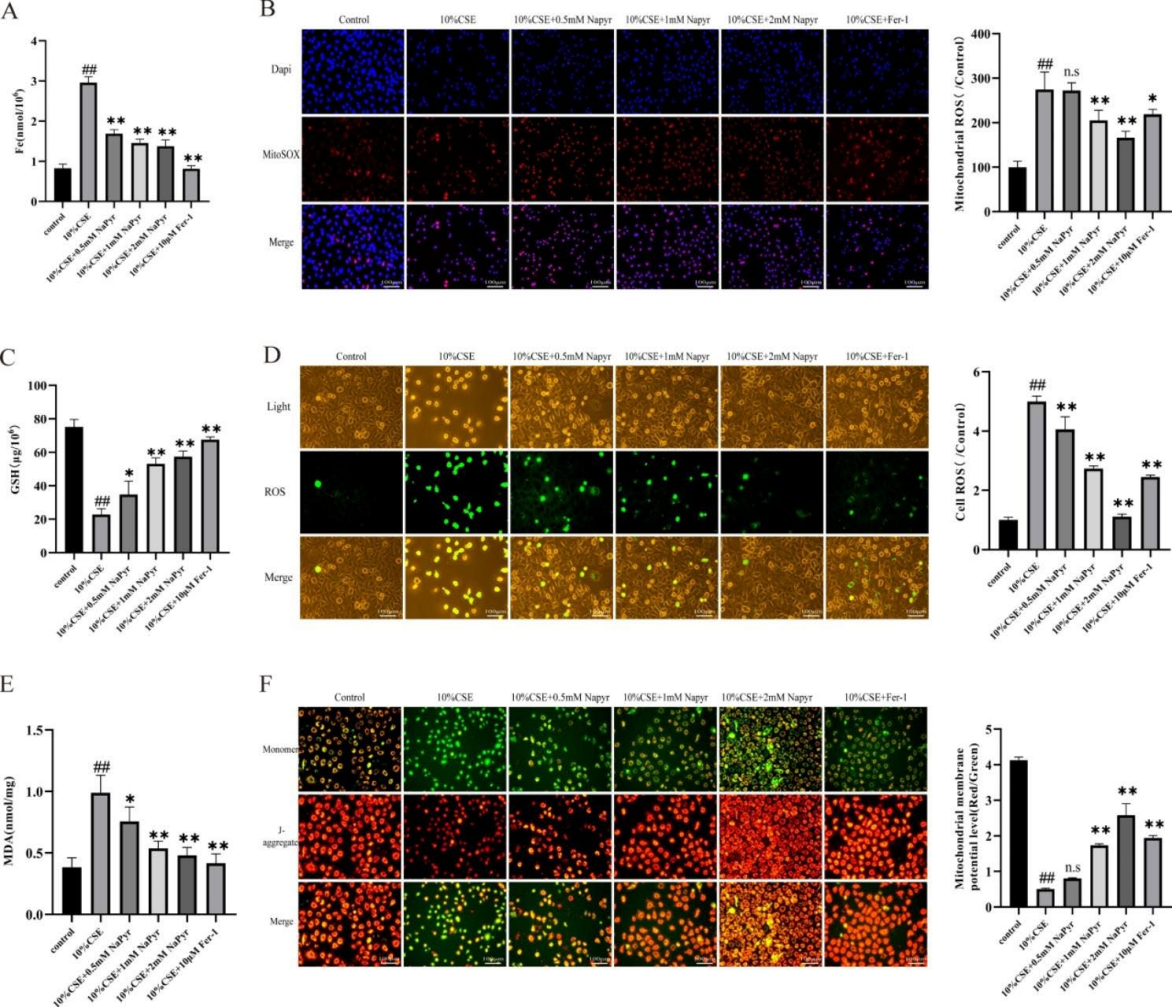
NaPyr inhibits CSE-induced ferroptosis in A549 cells. (**A**) Total iron levels in A549. (n = 4;^##^P < 0.01 vs. Control;^**^p < 0.01 vs. CSE) (**B**) The fluorescence intensity of MitoSOX in A549 was observed by fluorescence microscope. Scale bar = 100 μm (n = 3;^##^P < 0.01 vs. Control;^*^P < 0.05,^**^p < 0.01 vs. CSE) (**C**) GSH contents in different experimental groups. (n = 4;^##^P < 0.01 vs. Control;^*^P < 0.05,^**^p < 0.01 vs. CSE) (**D**) Napyr pretreatment can reduce the generation of ROS induced by CSE. Scale bar = 100 μm (n = 3;^##^P < 0.01 vs. Control;^**^p < 0.01 vs. CSE) (**E**) MDA levels in A549 of different experimental groups. (n = 4;^##^P < 0.01 vs. Control;^*^P < 0.05,^**^p < 0.01 vs. CSE) (**F**) The effect of CSE on MMP in A549 and the pretreatment effect of Napyr. Scale bar = 100 μm (n = 3;^##^P < 0.01 vs. Control;^**^p < 0.01 vs. CSE)

We next examined whether NaPyr exerts its protective effect on alveolar epithelial cells by regulating ferroptosis. Accordingly, CSE (10%)-induced A549 cells were treated with NaPyr and ferroptosis inhibitor Fer-1. Ferroptosis is characterized by cell death caused by iron accumulation and iron dependency. On measuring the intracellular iron concentration, we found that CSE could significantly increase the iron concentration, and both NaPyr and Fer-1 could significantly decrease the CSE-induced increase in iron concentration (Fig. 2A). MDA and GSH are two important antioxidant system components in vivo. In addition, ferroptosis is characterized by elevated MDA levels and reduced GSH concentration. Herein, CSE increased MDA levels while decreasing GSH levels in A549 cells, and these changes were significantly reversed by NaPyr or Fer-1 treatment (Fig. 2C and E). Elevated intracellular mitochondrial superoxide and ROS levels are key features of ferroptosis onset. CSE-induced A549 cells displayed significantly increased intra-mitochondrial superoxide and ROS levels, along with decreased mitochondrial membrane potential; treatment with either NaPyr or Fer-1 could rescue these changes (Fig. 2B, D, and F). Overall, these results suggested that NaPyr could inhibit CSE-induced ferroptosis in A549 cells.

3. NaPyr inhibits CSE-induced ferroptosis in BEAS-2B Cells.

Next, we determined whether NaPyr exerts its protective effects on bronchial epithelial cells by regulating ferroptosis. We treated CSE (5%)-induced BEAS-2B cells with NaPyr and ferroptosis inhibitor Fer-1 and measured the intracellular iron concentration. CSE induction significantly increased the iron concentration, and both NaPyr and Fer-1 could significantly reduce the CSE-induced increase in iron concentration (Fig. 2A). Pretreatment with NaPyr decreased ROS and MDA levels and increased GSH levels in alveolar and bronchial epithelial cells (Fig. 2C, D, and E), thereby revealing the role of NaPyr in improving CSE-induced oxidative damage. Cellular mitochondrial damage is a key feature of ferroptosis onset. CSE-induced BEAS-2B cells displayed significantly elevated intra-mitochondrial superoxide levels and reduced mitochondrial membrane potential; pretreatment with either NaPyr or Fer-1 could rescue these changes (Fig. 2B and F). Overall, these results suggested that NaPyr could inhibit CSE-induced ferroptosis in BEAS-2B cells (Fig. 4).

**Fig. 3:**
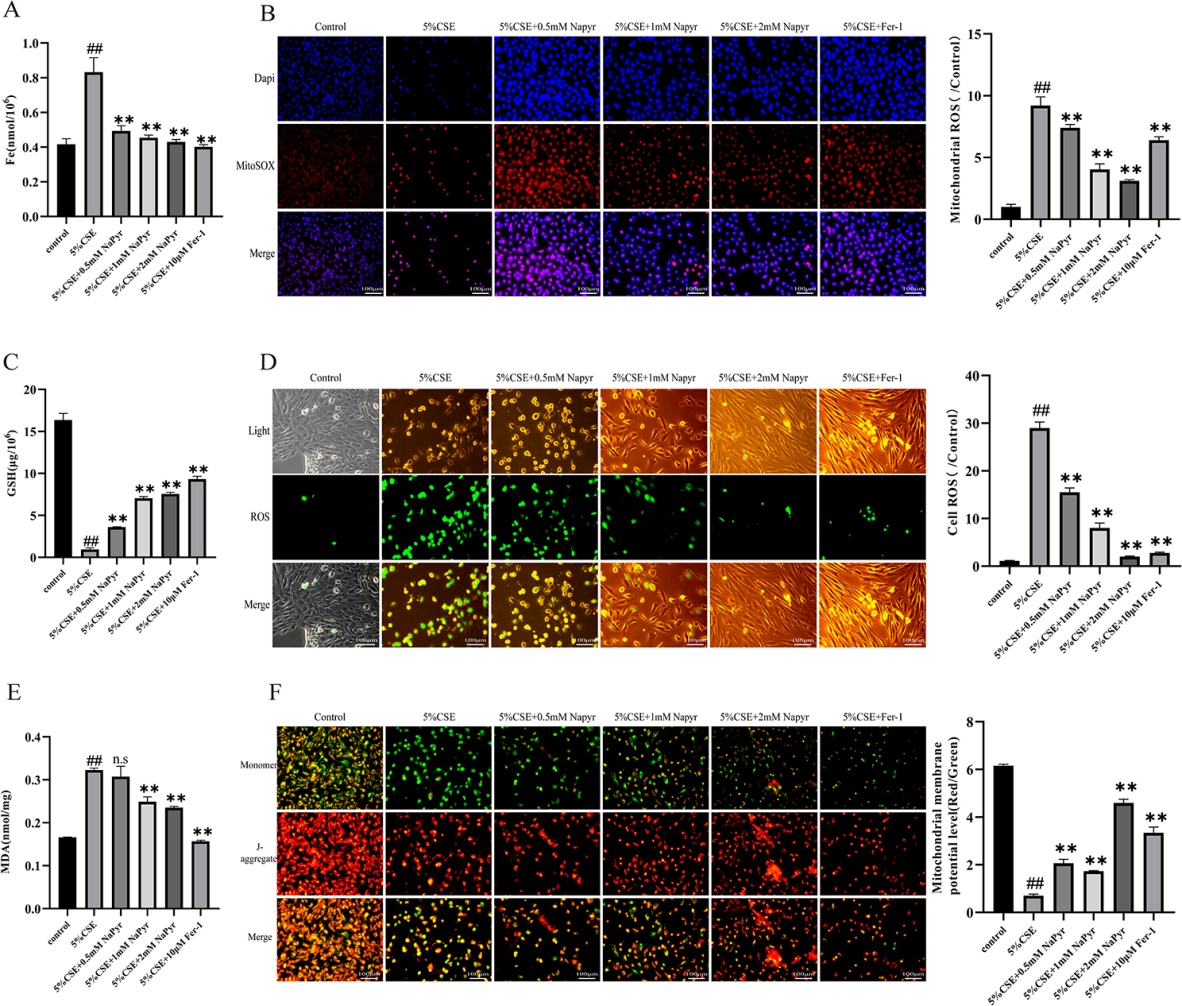
NaPyr inhibits CSE-induced ferroptosis in A549 cells. (**A**) Total Fe content of BEAS-2B cells in each group after CSE induction. (n = 4;^##^P < 0.01 vs. Control; ^**^p < 0.01 vs. CSE) (**B**) The fluorescence intensity of MitoSOX in BEAS-2B was observed by fluorescence microscope. Scale bar = 100 μm (n = 3;^##^P < 0.01 vs. Control;^**^p < 0.01 vs. CSE) (**C**) GSH content in BEAS-2B after CSE stimulation and the intervention of NaPyr pretreatment. (n = 4;^##^P < 0.01 vs. Control;^*^P < 0.05,^**^p < 0.01 vs. CSE) (**D**) Napyr pretreatment can reduce the generation of ROS induced by CSE. Scale bar = 100 μm (n = 3;^##^P < 0.01 vs. Control;^**^p < 0.01 vs. CSE) (**E**) MDA levels in BEAS-2B of different experimental groups. (n = 4;^##^P < 0.01 vs. Control;^**^p < 0.01 vs. CSE) (**F**) The effect of CSE on MMP in BEAS-2B and the pretreatment effect of Napyr. Scale bar = 100 μm (n = 3;^##^P < 0.01 vs. Control;^**^p < 0.01 vs. CSE)

4. NaPyr regulates CSE-induced ferroptosis in A549 and BEAS-2B cells via the GPX4/Nrf2 axis.

**Fig. 4:**
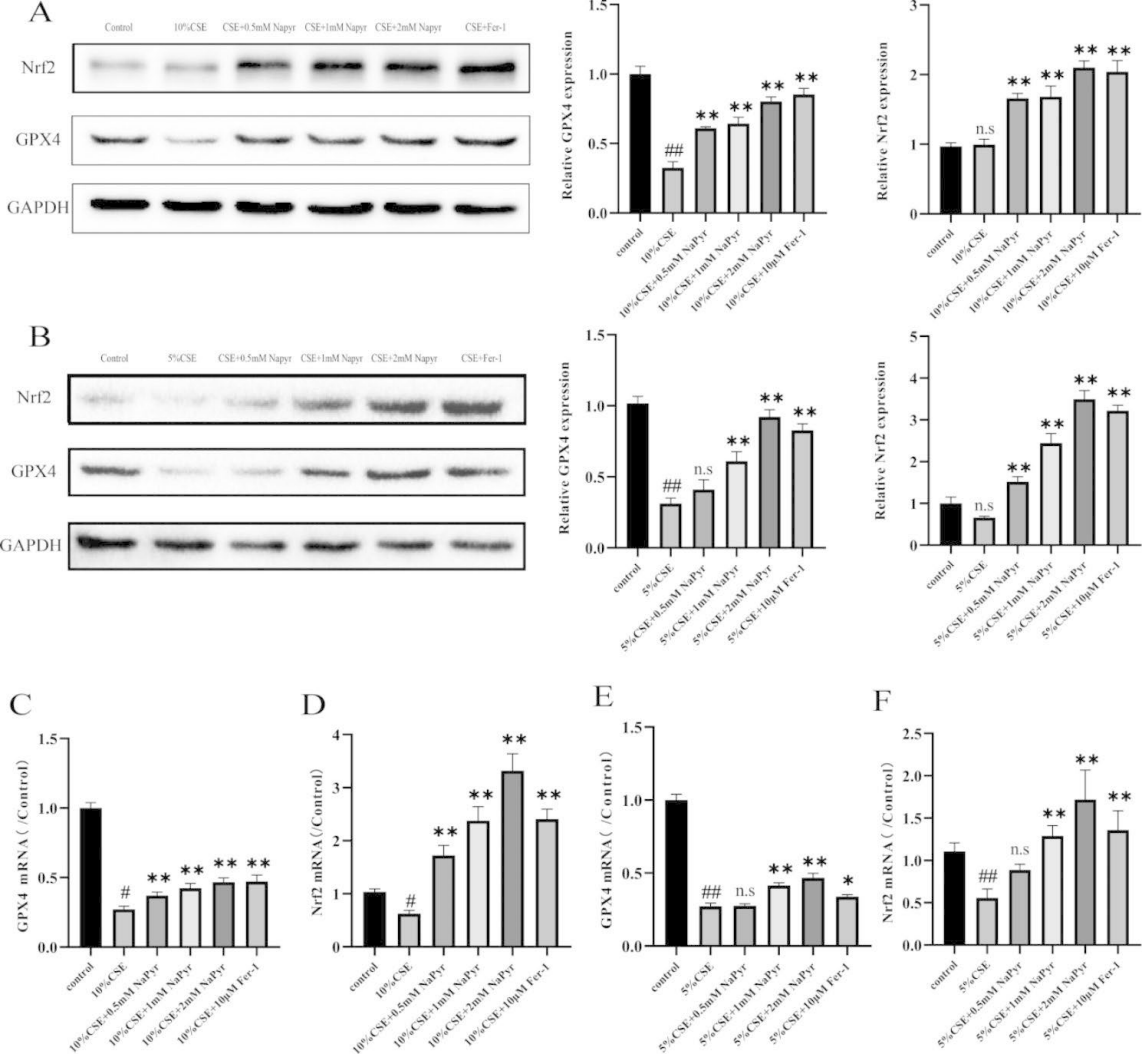
NaPyr regulates CSE-induced ferroptosis in A549 and BEAS-2B cells through the GPX4/Nrf2 pathway. (**A**) Protein expression levels of Nrf2 and GPX4 of A549 protein expression was measured by western blotting. (n = 3;^##^P < 0.01 vs. Control; ^**^p < 0.01 vs. CSE) (**B**) Protein expression levels of Nrf2 and GPX4 of BEAS-2B protein expression was measured by western blotting. (n = 3;^##^P < 0.01 vs. Control; ^**^p < 0.01 vs. CSE) (**C**) mRNA expression of GPX4 in A549 measured with qPCR. (n = 3;^#^P < 0.05 vs. Control; ^**^p < 0.01 vs. CSE) (**D**) mRNA expression of Nrf2 in A549 measured with qPCR. (n = 3;^#^P < 0.05 vs. Control; ^**^p < 0.01 vs. CSE) (**E**) mRNA expression of GPX4 in A549 measured with qPCR. (n = 3;^##^P < 0.01 vs. Control;^*^P < 0.05,^**^p < 0.01 vs. CSE) (**F**) mRNA expression of Nrf2 in BEAS-2B measured with qPCR. (n = 3;^##^P < 0.01 vs. Control; ^**^p < 0.01 vs. CSE)

It has been reported that NaPyr could reverse CSE-induced ferroptosis. To clarify the mechanism through which NaPyr counteracts CSE-induced cell damage, we examined intracellular ferroptosis-related proteins. We found that NaPyr regulated the GPX4/Nrf2 axis to inhibit ferroptosis in the CSE-induced COPD model, as evidenced by a significant CSE-induced decrease in the expression level of the ferroptosis-related protein GPX4 and a mild decrease in the level of simultaneously-produced ROS-activated Nrf2. Different NaPyr concentrations significantly reversed the CSE-mediated effects, with expression levels of ferroptosis-related proteins Nrf2 and GPX4 exhibiting an increasing trend in a NaPyr concentration-dependent manner (Fig. 4A and B). In addition, NaPyr treatment significantly increased the expression of Nrf2 and GPX4 at the mRNA level (Fig. 4C-F). These results indicated that NaPyr inhibited CSE-induced ferroptosis in alveolar and bronchial epithelial cells by activating the GPX4/Nrf2 axis.

5. NaPyr inhibits CSE-induced inflammatory damage in A549 and BEAS-2B cells by regulating COX2 expression.

**Fig. 5:**
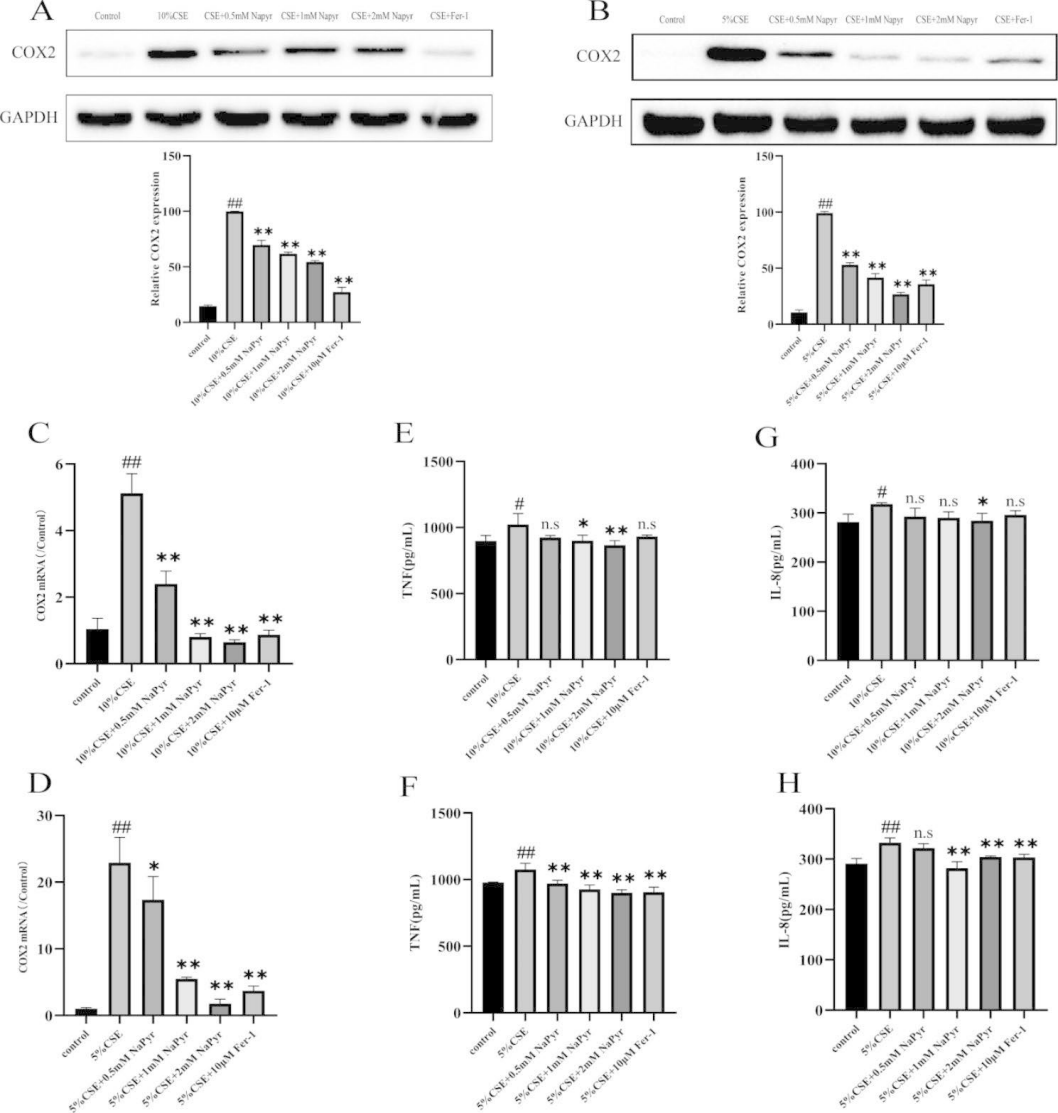
(**A**) Protein expression levels of COX2 of A549 protein expression was measured by western blotting.(n = 3;^##^P < 0.01 vs. Control; ^**^p < 0.01 vs. CSE) (**B**) Protein expression levels of COX2 of BEAS-2B protein expression was measured by western blotting. (n = 3;^##^P < 0.01 vs. Control; ^**^p < 0.01 vs. CSE) (**C**) mRNA expression of COX2 in A549 measured with qPCR. (n = 3;^##^P < 0.01 vs. Control; ^**^p < 0.01 vs. CSE) (**D**) mRNA expression of COX2 in BEAS-2B measured with qPCR. (n = 3;^##^P < 0.01 vs. Control; ^*^P < 0.05;^**^p < 0.01 vs. CSE) (**E**) The levels of TNF from A549 measured by ELISA assay. (n = 3;^#^P < 0.05 vs. Control; ^*^P < 0.05,^**^p < 0.01 vs. CSE) (**F**) The levels of TNF from BEAS-2B measured by ELISA assay. (n = 3;^##^P < 0.01 vs. Control; ^**^p < 0.01 vs. CSE) (**G**) The levels of IL-8 from A549 measured by ELISA assay. (n = 3;^#^P < 0.05 vs. Control; ^*^p < 0.05 vs. CSE) (H)The levels of IL-8 from BEAS-2B measured by ELISA assay. (n = 3;^##^P < 0.01 vs. Control; ^**^p < 0.01 vs. CSE)

NaPyr exerts antioxidant and anti-inflammatory effects and has potential therapeutic efficacy in inflammatory diseases[14]. COX2 is a specific ferroptosis marker and stimulates the production of inflammatory factors in the airways. COX2 expression was found to be significantly increased in patients with COPD, accompanied by substantial airway inflammation. After CSE induction of A549 and BEAS-2B cells, intracellular COX2 protein levels, as well as mRNA expression, were increased, and administration of NaPyr significantly decreased COX2 expression (Fig. 5A-D). Furthermore, CSE stimulation significantly increased levels of pro-inflammatory cytokines IL-8 and TNF, and NaPyr significantly suppressed these levels. Taken together, these results suggested that NaPyr downregulates the COX2 pathway and reduces the secretion of inflammatory factors IL-8 and TNF, thereby inhibiting CSE-induced inflammatory damages in alveolar and bronchial epithelial cells.

## Discussion

COPD is currently the third-leading cause of mortality worldwide, and its high prevalence, disability, and mortality rates induce marked economic and societal burdens in China. Smoking is the most important risk factor for COPD, asthma, and other lung diseases. Cigarette smoke and other harmful particles enter the lungs through the airways and affect human bronchial and alveolar epithelial cells; these particles induce inflammation and peroxidative stress in airways and alveolar epithelial cells, resulting in pulmonary dysfunction and COPD onset[23, 24]. Ferroptosis, a recently discovered type of cell death, is characterized by excessive iron accumulation and lipid peroxidation. Growing evidence suggests that this iron-dependent form of non-apoptotic cell death is associated with cigarette smoke-induced COPD pathogenesis[25]. Considering various therapies for COPD, NaPyr is an antioxidant naturally present in the body. Exogenously administered sodium pyruvate exerts various biological effects, including antioxidant and anti-inflammatory effects, affording a novel therapy for COPD. The present study focused on the role and mechanism of NaPyr in regulating CSE-induced ferroptosis in alveolar and bronchial epithelial cells. Herein, our findings revealed that NaPyr could improve ferroptosis-mediated alveolar and bronchial epithelial cell damage by activating the GPX4/Nrf2 axis. In addition, NaPyr could reduce the production of alveolar and bronchial epithelial cell inflammatory factors TNF and IL-8 by modulating COX2 expression.

Regulated cell death in the alveolar and bronchial epithelial cells reportedly plays an important role in COPD pathogenesis. Considering patients with COPD, alveolar and bronchial epithelial cell death has been associated with smoking[26]. Herein, we stimulated alveolar and bronchial epithelial cells using CSE and found that CSE significantly reduced A549 and BEAS-2B cell activity. In the present study, treatment with the ferroptosis inhibitor Fer-1 partially increased CSE-induced A549 and BEAS-2B cell activity. This finding suggests that CSE induces cell injury and death, including ferroptosis. Previous studies have revealed that NaPyr can exert a protective effect against multi-organ dysfunction[27]. In the present study, NaPyr treatment significantly increased cell viability in a concentration-dependent manner, suggesting that NaPyr could reduce alveolar and bronchial epithelial cell injury. Erastin is a classic ferroptosis inducer. NaPyr could rescue the reduced activity of A549 and BEAS-2B cells caused by erastin stimulation, suggesting that NaPyr combats ferroptosis in alveolar and bronchial epithelial cells to afford a protective effect.

Cigarettes contain several oxidizing substances. Oxidative stress induced by intracellular accumulation of harmful free radicals is considered a major factor in cigarette smoke-induced pathological changes[28]. Reportedly, CSE can induce ferroptosis in lung epithelial cells and HBECs, thereby acting as a key factor contributing to COPD[29]. Smokers are known to exhibit abnormally high levels of iron accumulation in the lung. Large amounts of intracellular free iron can directly produce substantial amounts of ROS via the Fenton reaction, including mitochondrial ROS and lipid peroxidation end products. Herein, NaPyr pretreatment significantly decreased CSE-induced intracellular free iron accumulation. In addition, mitochondria are a major source and target site of intracellular ROS, and cigarette smoke-induced mitochondria were found to be severely damaged in the airway epithelial cells of rats with COPD[30]. We detected an increased mitochondrial MitoROS concentration and decreased MMP levels in CSE-stimulated alveolar and bronchial epithelial cells, suggesting that CSE can induce mitochondrial oxidative stress damage in alveolar and bronchial epithelial cells. NaPyr intervention could significantly improve the changes in mitochondrial superoxide indicators, suggesting that NaPyr can reduce mitochondrial peroxidative stress. Previous studies have confirmed that pyruvate is an efficient ROS scavenger[19], consistent with the findings of the present study. NaPyr treatment reduced ROS and MDA levels and enhanced GSH levels in alveolar and bronchial epithelial cells, suggesting that NaPyr could improve CSE-induced oxidative damage. Thus, NaPyr exerts its therapeutic effects against COPD by suppressing ferroptosis in alveolar and bronchial epithelial cells.

Furthermore, our results demonstrate that NaPyr could improve ferroptosis in CSE-mediated lung epithelial and bronchial epithelial cells by activating the GPX4/Nrf2 axis. GPX4 is a type of selenoprotein glutathione peroxidase that can decrease lipid peroxide-mediated toxicity, whereas Nrf2 is an important component of the endogenous antioxidant pathway. Mouse models of COPD demonstrate reduced GPX4 expression and enhanced Nrf2 expression, both key ferroptosis regulators[31–34]. It has been reported that activating the GPX4/Nrf2 axis is the key to combating ferroptosis. Additionally, COX2 is a known ferroptosis marker. In the present study, NaPyr decreased COX2 levels in CSE-induced alveolar and bronchial epithelial cells and increased gene and protein levels of GPX4 and Nrf2. Therefore, NaPyr may inhibit ferroptosis in lung epithelial and bronchial epithelial cells, and the underlying mechanism might be related to the regulation of the GPX4/Nrf2 axis.

## Conclusions

The findings of our study revealed that NaPyr could combat CSE-induced ferroptosis via the GPX4/Nrf2 axis and could reduce the production of inflammatory factors such as TNF and IL-8 in alveolar and bronchial epithelial cells. Accordingly, this study provides substantial evidence regarding the NaPyr-mediated COPD preventive and therapeutic effects.

## Data Availability

All data used during the current study are available from the corresponding authors on reasonable request.
