# Correction: Sodium pyruvate exerts protective effects against cigarette smoke extract-induced ferroptosis in alveolar and bronchial epithelial cells through the GPX4/Nrf2 axis

**DOI:** 10.1186/s12950-025-00436-y

**Published:** 2025-02-17

**Authors:** Ziwen Zhao, Zhao Xu, Jingwen Chang, Liwei He, Zijin Zhang, Xiaoyu Song, Xianbang Hou, Fangtian Fan, Zhijun Jiang

**Affiliations:** 1 School of Pharmacy, Nanjing University of Chinese Medicine, 138 Xianlin Avenue, Nanjing 21, Taizhou, Jiangsu 210023 China; 2 Jiangsu Changtai Pharmaceutical Co., Ltd, Taizhou, Jiangsu 225300 China; 3 Anhui Engineering Technology Research Center of Biochemical Pharmaceuticals, School of Pharmacy, Bengbu Medical College, 2600 Donghai Avenue, Bengbu, Anhui 233003 China; 4 School of Pharmacy, Nanjing University of Chinese Medicine, Nanjing, 210023 China

**Correction to: Zhao et al. Journal of Inflammation (2023) 20:28**

10.1186/s12950-023-00347-w.

After the publication of the original article, the authors reported errors in Fig. 3B due to a mistake in copying and pasting during the figure assembly process and oversight in proofreading.

The error does not affect the conclusion. The authors have now corrected the image as shown below. The authors apologize for any inconvenience caused to the journal and its readers.

**Incorrect Fig. 3**.


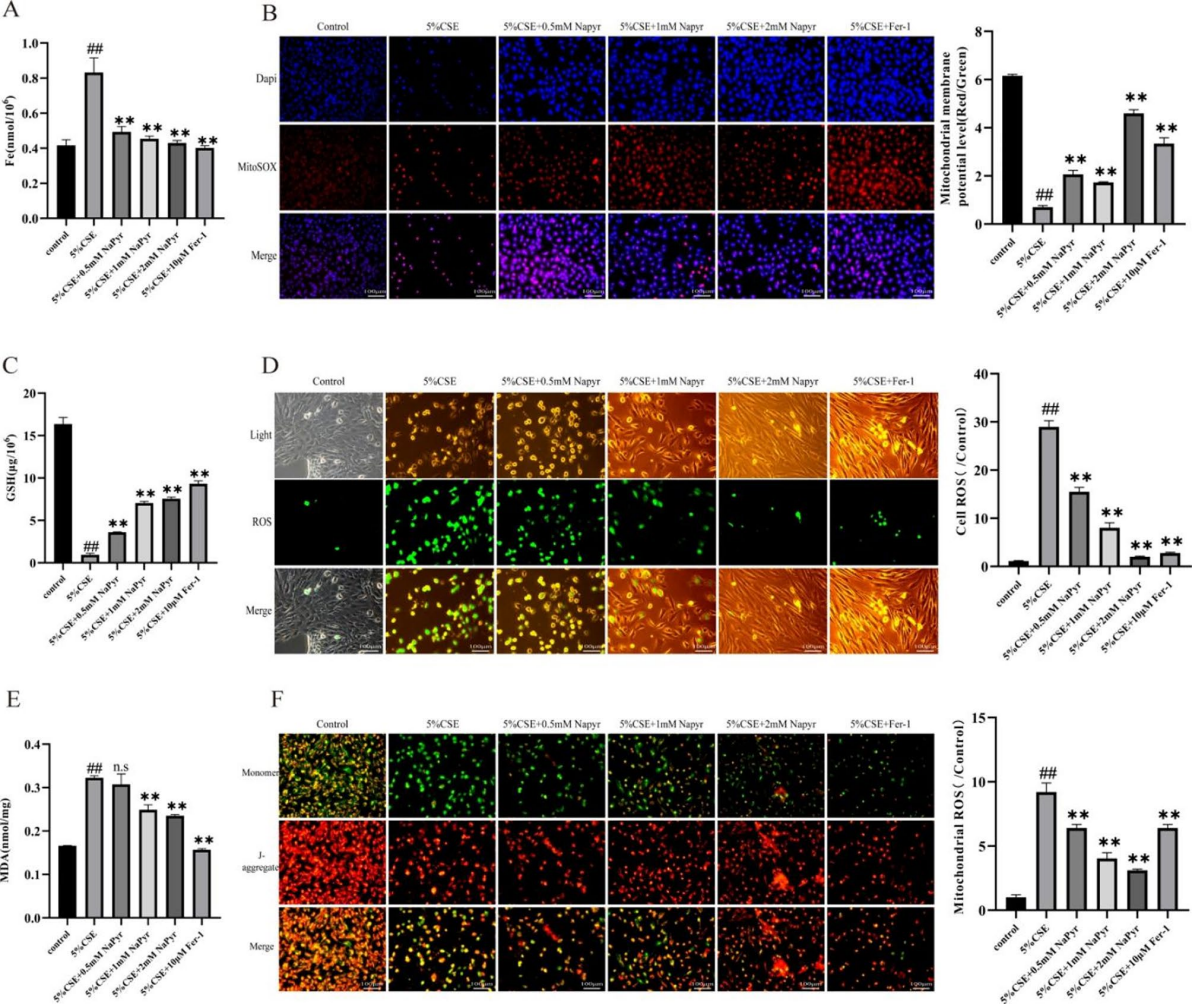


**Correct Fig. 3**.


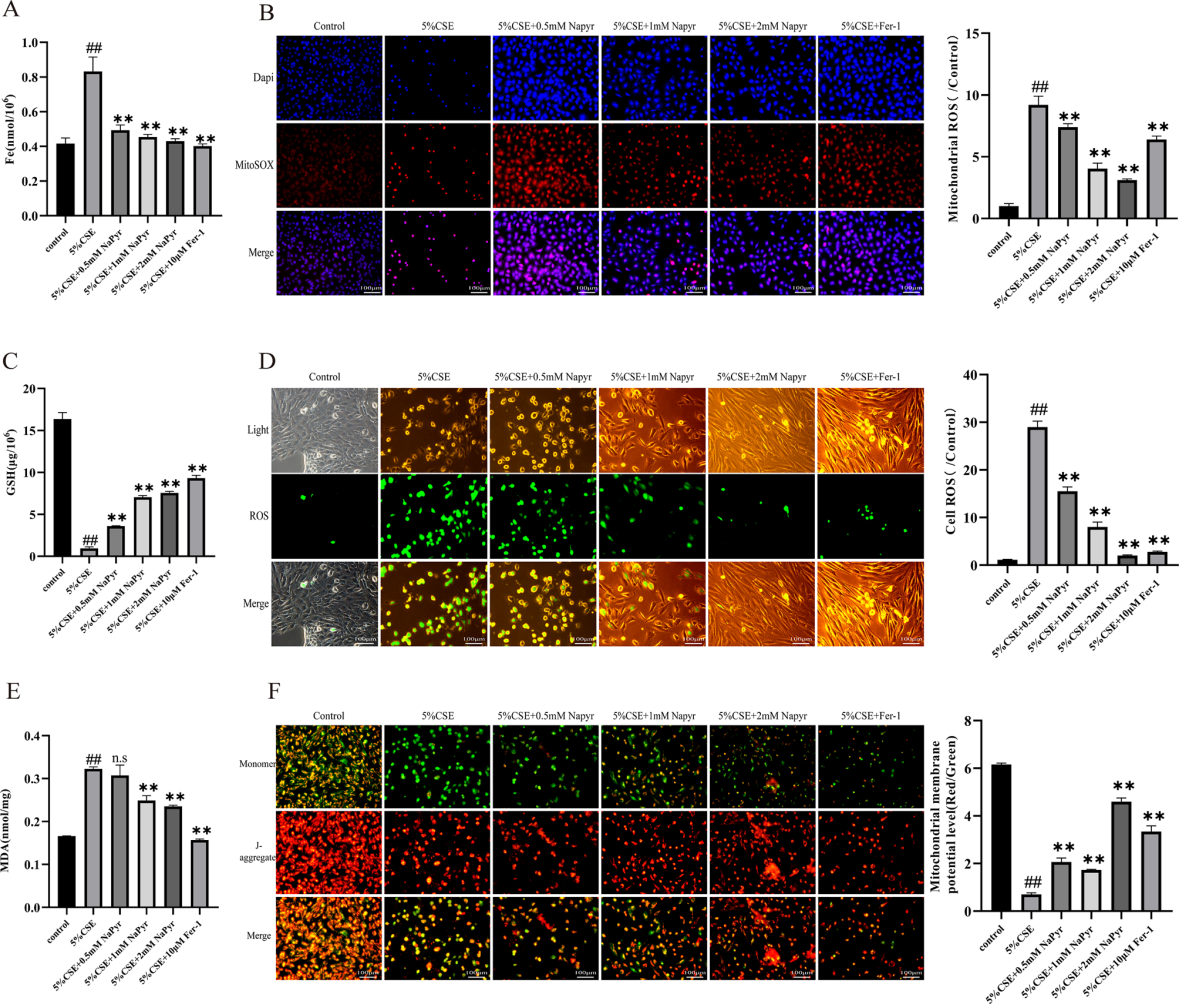


The original article has been corrected.

